# Enteric Fever in South Africa

**Published:** 1900-11-17

**Authors:** 


### ENTERIC FEVER IN SOUTH AFRICA.

An important article appears in the *British Medical Journal* from the pen of Dr. Howard Tooth, assistant physician St. Bartholomew's Hospital, and lately physician to the Portland Hospital, in which he discusses the various modes by which the infection of enteric fever was spread in South Africa. These he enumerates as water, dust, and flies.

In regard to water he seems to have but little faith in the various methods adopted and recommended by the "arm-chair hygienist." Standing in the way of the efficiency of any of these measures, there is, firstly, the apparently invincible ignorance and apathy of the soldier about all such matters, and next comes the fact that thirst is the most imperious of appetites. "Boiling and filtering take time, and a raging thirst will not wait, but will be satisfied regardless of consequences." Speaking of the several methods employed for purifying the water, he says that when water was boiled it took a long time to cool, while even after all it was not very palatable, while the filters quickly became blocked, and, notwithstanding all that was done in the way of purifying the water, men from the very regiments which took these precautions could be seen all day long filling their water bottles at the Modder.

The next mode of spread to which he refers is that by whirlwinds and dust. Besides whirlwinds by which pieces of paper and other articles were carried up an astonishing height into the air, the whole plain was sometimes swept by gales which would bring clouds of dust from one end of the camp to the other. While he was at the Modder the food was rarely free from grit, and under such circumstances it would seem almost impossible to prevent its contamination. Lastly, the flies. These were indistinguishable from ordinary house flies, but their multitude was enormous, and, curiously, they seemed to have a preference for the enteric cases. "It was striking to see how in a tentful of sick the flies preferred the enteric fever cases to the exclusion of others, dysentery or sunstroke, equally if not more ill."

